# Bach Mai Procedure for complete mesocolic excision, central vascular ligation, and D3 lymphadenectomy in total laparoscopic right hemicolectomy: a prospective study

**DOI:** 10.1186/s12957-023-03026-5

**Published:** 2023-05-05

**Authors:** Ngoc Hung Nguyen, Xuan Vinh Vu, Vu Quang Nguyen, The Hiep Nguyen, Huy Du Nguyen, Tuan Hiep Luong, Thanh Khiem Nguyen, Ham Hoi Nguyen

**Affiliations:** 1 Department of Gastrointestinal and Hepato-Pancreato-Biliary Surgery, Bach Mai Hospital, Hanoi, Vietnam; 2 Department of Surgery, Hanoi Medical University, Hanoi, Vietnam

**Keywords:** Total laparoscopic right hemicolectomy, Complete mesocolic excision, Central vascular ligation, D3 lymphadenectomy

## Abstract

**Purpose:**

Total laparoscopic right hemicolectomy with complete mesocolic excision (CME), central vascular ligation (CVL), and D3 lymphadenectomy is still the most challenging colon procedures for gastrointestinal surgeons. We herein report the technical details and our preliminary experience of Bach Mai Procedure — a novel-combining (cranial, medial to lateral, and caudal) approach with early resection of the terminal ileum.

**Methods:**

The dissection stage was central vascular isolation and ligation by a combined multiple approaches in the following four steps: cranial approach, dissecting along the inferior aspect of pancreatic isthmus to reveal the middle colic vessels and the anterior aspect of the superior mesentery vein and then exposed the right gastroepiploic vein and the trunk of Henle; medial-to-lateral approach, exposing the surgical axis — the superior mesenteric vascular axis and then early resection of the terminal ileum to open the dissection from the bottom up; and caudal approach, radical ligation of the ileocecal artery and right colic artery (central vascular ligation), lymph node dissection (D3 lymphadenectomy), and resecting the Toldt fascia of the colon to release the entire right colon from the abdominal wall.

**Results:**

In 12 months, there were 32 cases of primary right-sided colon malignancies that have undergone tLRH_D3, CME/CVL_ based on the Bach Mai Procedure. In 3 cases (9.4%), the tumor site was hepatic flexure. The median of lymph node number (LNN) was 38, with the maximum number which was 101. No serious postoperative complications (grade 3 or higher) neither inhospital mortality was detected.

**Conclusion:**

This Bach Mai procedure, a novel-combining approach with early resection of the terminal ileum, is technically feasible and safe for tLRH_D3, CME/CVL_. Further investigations and follow-up must be proceeded to evaluate the long-term outcomes of our technique.

**Supplementary Information:**

The online version contains supplementary material available at 10.1186/s12957-023-03026-5.

## Introduction

Colorectal cancer (CRC) is the worldwide second most cause of cancer death in both sexes, and proximal colon, including right-sided colon (RSC) and transverse colon to the splenic flexure, accounts for the highest rate, about 40% of all cases [1, 2]. Right (standard or extended) hemicolectomy was the only curative treatment for the American Joint Committee on Cancer (AJCC) stages 1 to 3 right-sided colon cancer [3, 4]. The technique of traditional laparoscopic right hemicolectomy (LRH) has been popularized and performed routinely for decades. However, until now, RCC was still proved to be an independent risk factor for recurrence in stage 2 disease, with a worse 5-year overall survival (OS) for stages 2 and 3 disease compared to left-sided colon and rectal cancer [3, 5]. So, there have been several new approaches for LRH to accomplish the oncological criteria as well as minimize the rate of postoperative anastomotic leakage. They are complete mesocolic excision (CME) with central vascular ligation (CVL) and D3 lymphadenectomy.

The concept of CME and CVL were first introduced by Hohenberger et al. in 2009 and based on the same embryological and pathological principles of total mesorectal excision (TME) applied in rectal cancer [6]. CME included a sharp dissection of the retroperitoneal visceral plane and full exposing of the mesenteric root and the origin of the vessels feeding the tumor (the ileocolic vein, right colic vein, Henle trunk, and middle colic vein on their appearance from the SMV and the ileocolic artery, right colic artery, and middle colic artery on their emergence from the SMA), thus allowing performing a CVL. Since that, there have been several trials such as “RELARC” and COLD trials which were processed to evaluate the oncological treatment effects of CME against convenient resection [7, 8], and some cohort studies with large prospective data was revealed the superior aspects of CME on risk of recurrence after resection with acceptable postoperative complications comparing with non-CME [9, 10]. Recently, a systematic review was concluded a favor oncological and survival outcome without increasing postoperative complications for CME [11]. Despite laparoscopic right hemicolectomy was considered to be a beginner’s colorectal surgery procedure, laparoscopic right hemicolectomy with CME was still a challenging technique and should be proceed by experienced surgeons [12].

Otherwise, D3 lymphadenectomy for right-sided colon cancer has been a “standard” surgical protocol for Japanese right-sided colon cancer treatment [13]. According to the Japanese Society for Cancer of the Colon and Rectum (JSCCR), the definition of D3 lymphadenectomy was dissection of the main lymph nodes (along superior mesenteric vessels, or N3 region), intermediate lymph nodes (N2 region), and pericolic lymph nodes (N1 region) [13]. Total laparoscopic right hemicolectomy with CME/CVL and D3 lymphadenectomy, or tLRH_D3, CME/CVL_, is still the most challenging colon procedures for gastrointestinal surgeons. There were some approaches for CME/CVL and D3 lymphadenectomy in tLRH which have been developed recently; however, none of the approaches has shown outstanding advantages over other approaches. Most of them were concentrated in the CME/CVL concepts, and there was a few studies that combined D3 lymphadenectomy and complete mesocolic excision for LRH, but in these studies, the anastomosis was performed extracorporeally or not mentioned [14–16]. Herein, we have presented Bach Mai Procedure — a novel-combining (cranial, medial to lateral, and caudal) approach with early resection of the terminal ileum for tLRH_D3, CME/CVL_ with illustration of highlights video and pictures as well as short-term outcomes.

## Materials and methods

### Patients

This case series has been reported in line with the PROCESS Guideline at the end of the “Materials and methods” section (and include citation in the references section) [17] and was registered in accordance with the Declaration of Helsinki. After an Institution Review Board approval, all patients with a histologically proven diagnosis of malignancies arise from right-sided colon who underwent tLRH with CME, CVL, and D3 lymphadenectomy at the Department of Gastrointestinal and Hepato-Pancreato-Biliary Surgery, Bach Mai Hospital, between January 2022 and December 2022 (12 months) were prospectively enrolled. Preoperative diagnosis was histologically confirmed as malignancies and staging were classified based on the eighth edition of the AJCC/UICC system of malignancies which arise from right-sided colon [18].

### Selection criteria

All patients with a histologically proven diagnosis of malignancies arise from right-sided colon who underwent tLRH_D3, CME/CVL_ at the Department of Gastrointestinal and Hepato-Pancreato-Biliary Surgery, Bach Mai Hospital, between January 2022 and December 2022 (12 months) were prospectively enrolled.

### Data collection

The data collected included patient demographics, clinical data of patients (general information, disease’s characteristics), the characteristics of the technique (standard or extended tLRH, operative time, intraoperative blood loss, length of resected bowel, total harvested nodes, number of positive nodes), and the short-term results (postoperative complications, rate of mortality, and length of postoperative stay).

### Statistical methods

Continuous variables are presented as a mean with standard deviation, or as median with range or interquartile range, depending on the distribution of the data. All statistical analyses were performed using SPSS, version 22.0 for Windows statistical software.

### Surgical technique

**Fig. 1:**
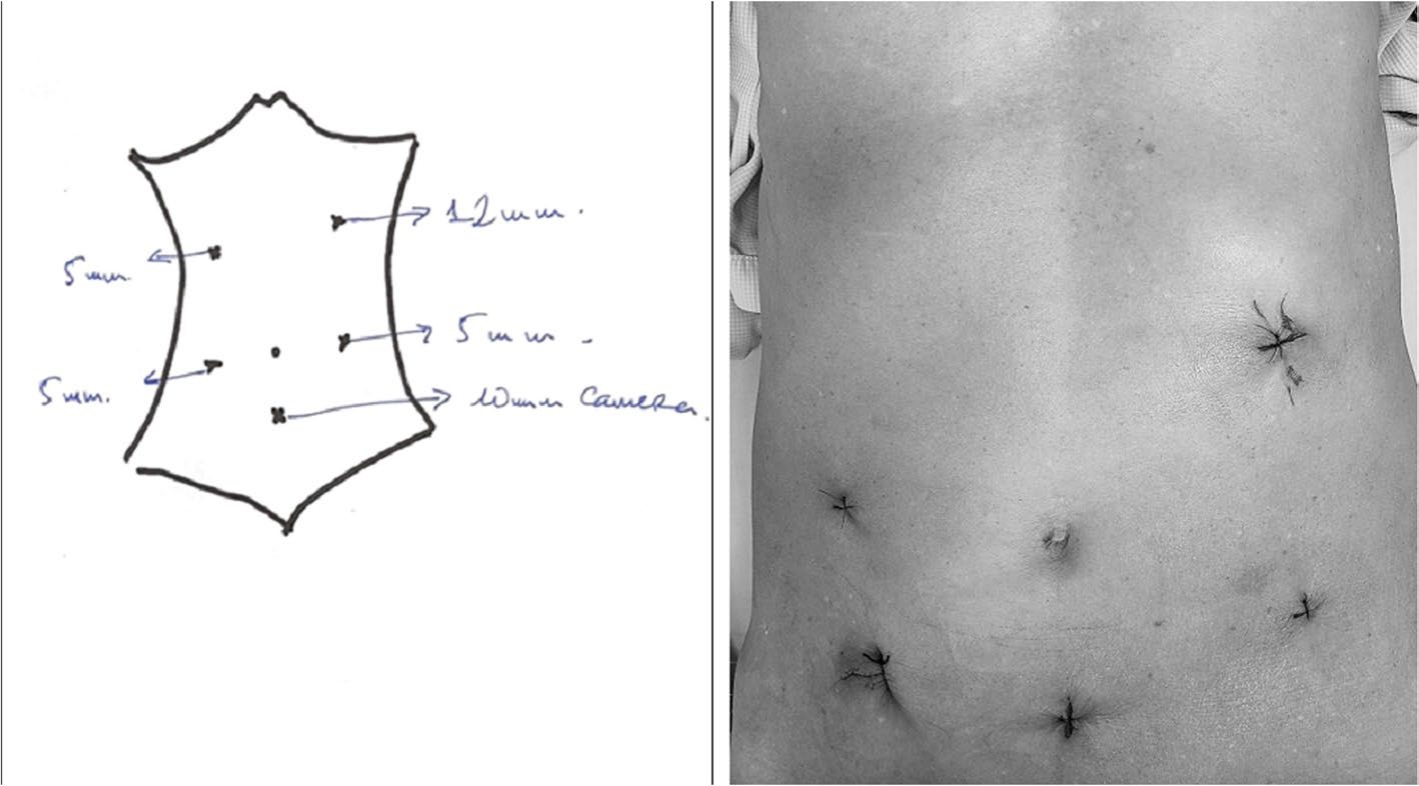
Trocar’s placement and the small opening across horizontal line on pubic bone or through the natural orifice (vagina) to collect the specimen

The patient was placed in the lithotomical position, with two legs lowered as much as possible to avoid affecting the operation of the surgeon. We use 5 trocars: one 10-mm trocar under the umbilicus about 4 cm for camera; on the left side, one 12-mm trocar at the intersection of transpyloric line and midclavicular line and one 5-mm trocars placed at the level compared to camera trocar’s placement; and two 5-mm trocars placed at the midclavicular line higher than 4 cm and in the level compared to camera trocar’s placement in the right side for laparoscopic instruments, respectively (Fig. 1). The surgeon stood on the left side of the patient, the first assistant stood on the right side, and the second assistant stood between two legs holding the camera. The monitor was located on the patient’s right side.

The procedure was divided into three steps.

**Fig. 2:**
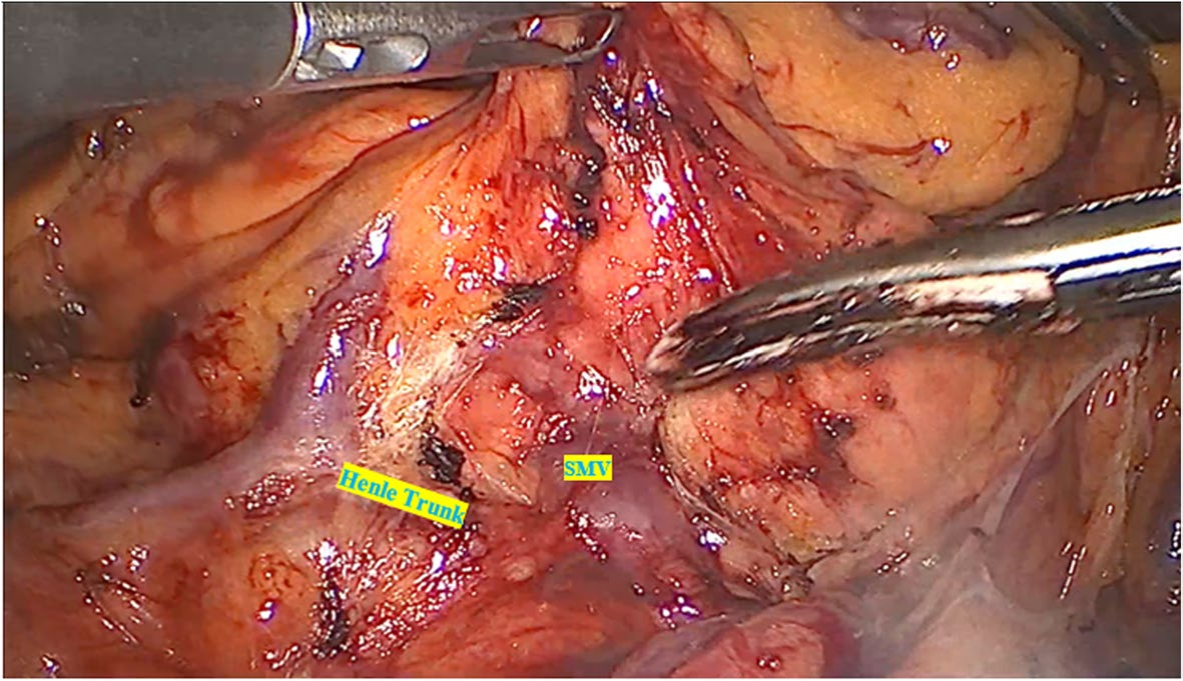
Dissected along the inferior aspect of pancreatic isthmus to reveal the anterior aspect of the superior mesentery vein (SMV) and the trunk of Henle

**Fig. 3:**
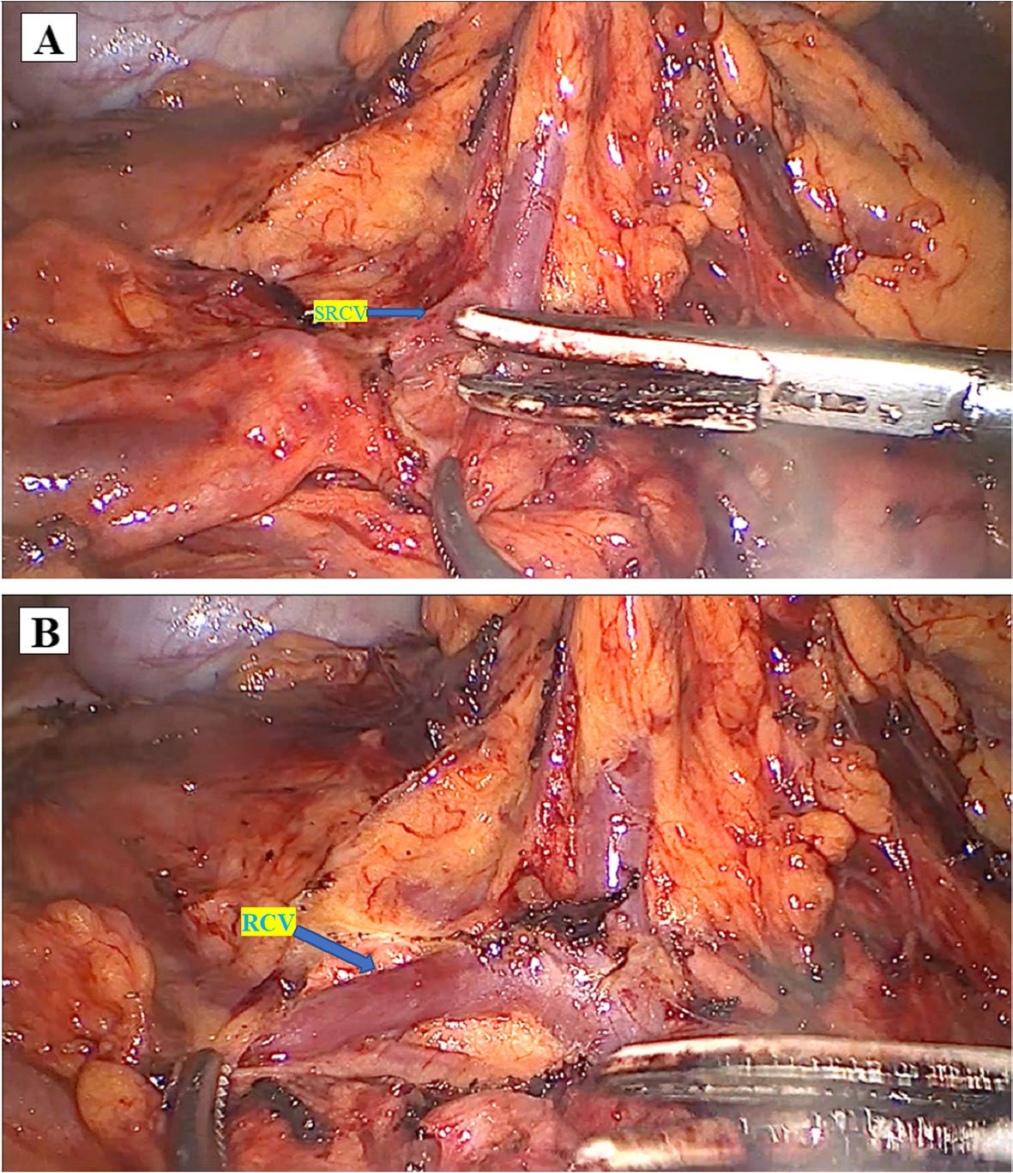
Ligated the right superior colic (**A**) and right colic veins (**B**)

- *Stage 1*: Evaluate the entire abdomen for liver and peritoneal metastases, and the possibility of laparoscopic right colectomy: low position, tilt left, and push the great omentum upward to the left to expose the right colon as much as possible. From terminal ileum to mid-transverse colon, assess tumor size and invasion and lymph node status.
- *Stage 2 (Dissection stage*): Release the ascending colon by a combined multiple approaches in the following four steps:

Step 1 (cranial approach): The patient was placed in a high head position and left side tilted. Firstly, opened the great omentum below the antrum to enter the omental bursa and detached the great omentum from transverse and the hepatic flexure colon to enter the omental bursa and reveal the duodenum and pancreatic head. Then, dissect along the inferior aspect of pancreatic isthmus to reveal the middle colic vessels and the anterior aspect of the superior mesentery vein, then exposed the right gastroepiploic vein and the trunk of Henle (Fig. 2), and then ligate the right superior colic and right colic veins (if there are drainage veins of the trunk of Henle) (Fig. 3); during dissection, simultaneously dissect the great omentum from the transverse mesocolon and colic hepatic flexure, dissect the transverse colic mesentery and a part of the right mesentery from the anterior surface of the pancreatoduodenal junction to enter the Fredet’s fascia, and when the right colic vein was revealed, then ligate this vein with LigaSure™ (Video 1).

**Fig. 4:**
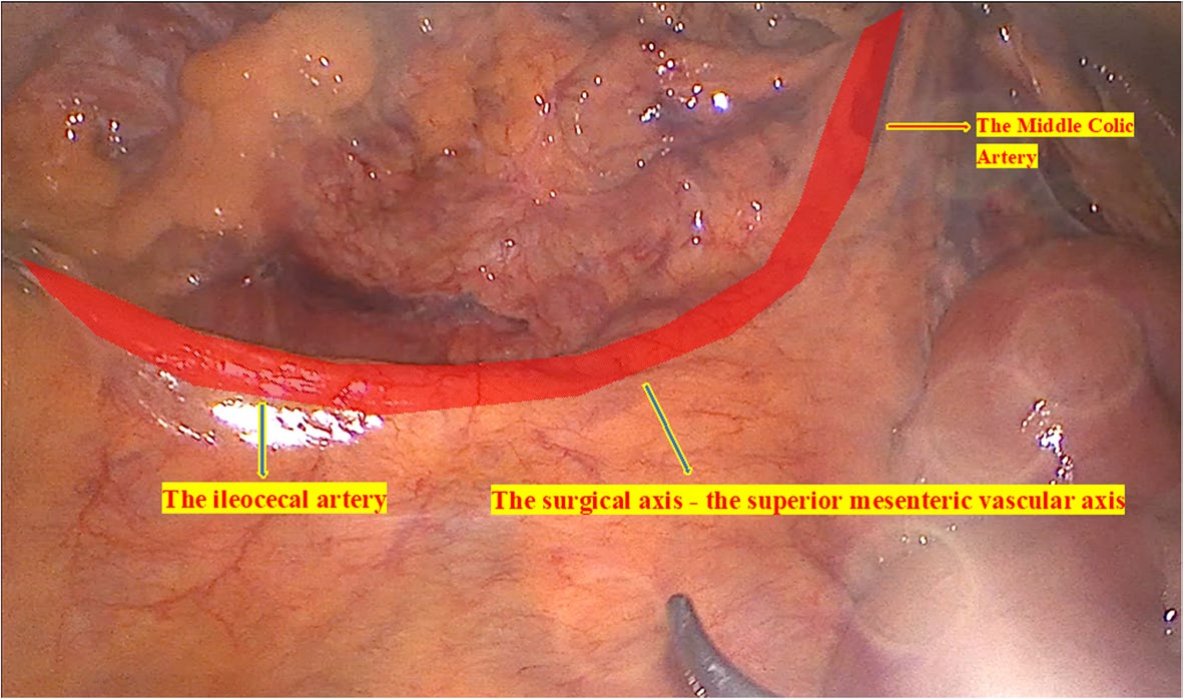
Exposed the surgical axis — the superior mesenteric vascular axis

**Fig. 5:**
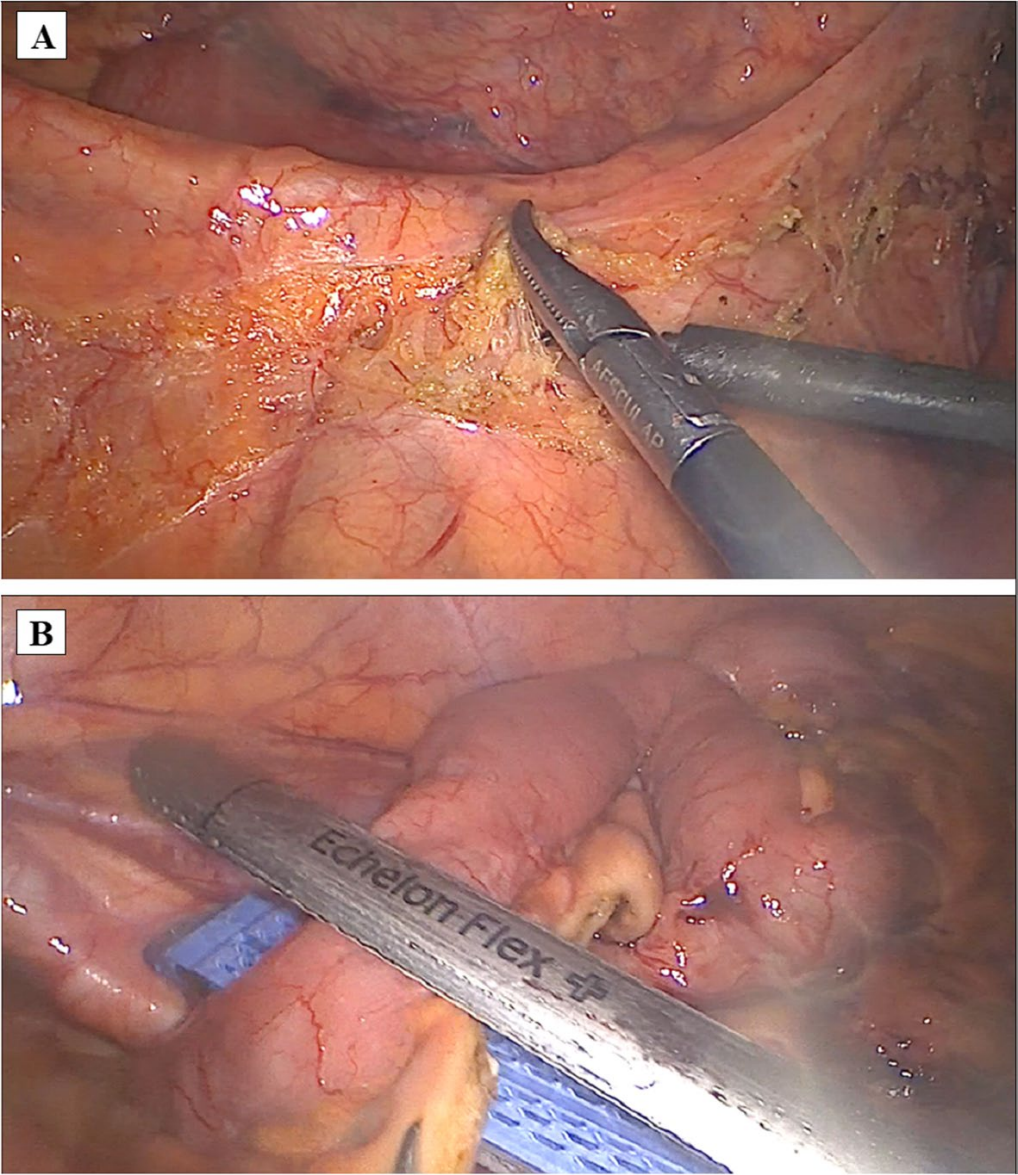
Open the mesenteric peritoneum along the left border of SMA from the medial to lateral sides, above the origin of the MCA, then through the origin of the ICA into the ileocecal fold of Treves, or avascular area of Treves, opening the fascia window (**A**). Early resection of the terminal ileum at this stage (**B**)

Step 2 (Medial-to-lateral approach): Exposed the surgical axis — the superior mesenteric vascular axis (Fig. 4). Used a nontraumatic pince to lift the middle colic, and ileocecal arteries — the beginning and end points of the surgical axis — will clearly show the superior mesenteric artery peritoneal fold (Video 1). Open the mesenteric peritoneum along the left border of this artery from the medial to lateral sides, above the origin of the middle colic artery (MCA), and then through the origin of the ileocecal artery (ICA), open the fascia window into the avascular area of Treves. There is early resection of the terminal ileum at this stage to open the dissection from the bottom up — this is one of the fundamental differences in the Bach Mai Procedure compared to other techniques, which favor D3 lymphadenectomy due to easy access to the origin of the vessel pedicles and to the posterior aspect of the superior mesenteric axis (Fig. 5). Dissect the connective tissue and the longitudinal lymph nodes anterior aspect of the superior mesenteric axis (Video 1).

**Fig. 6:**
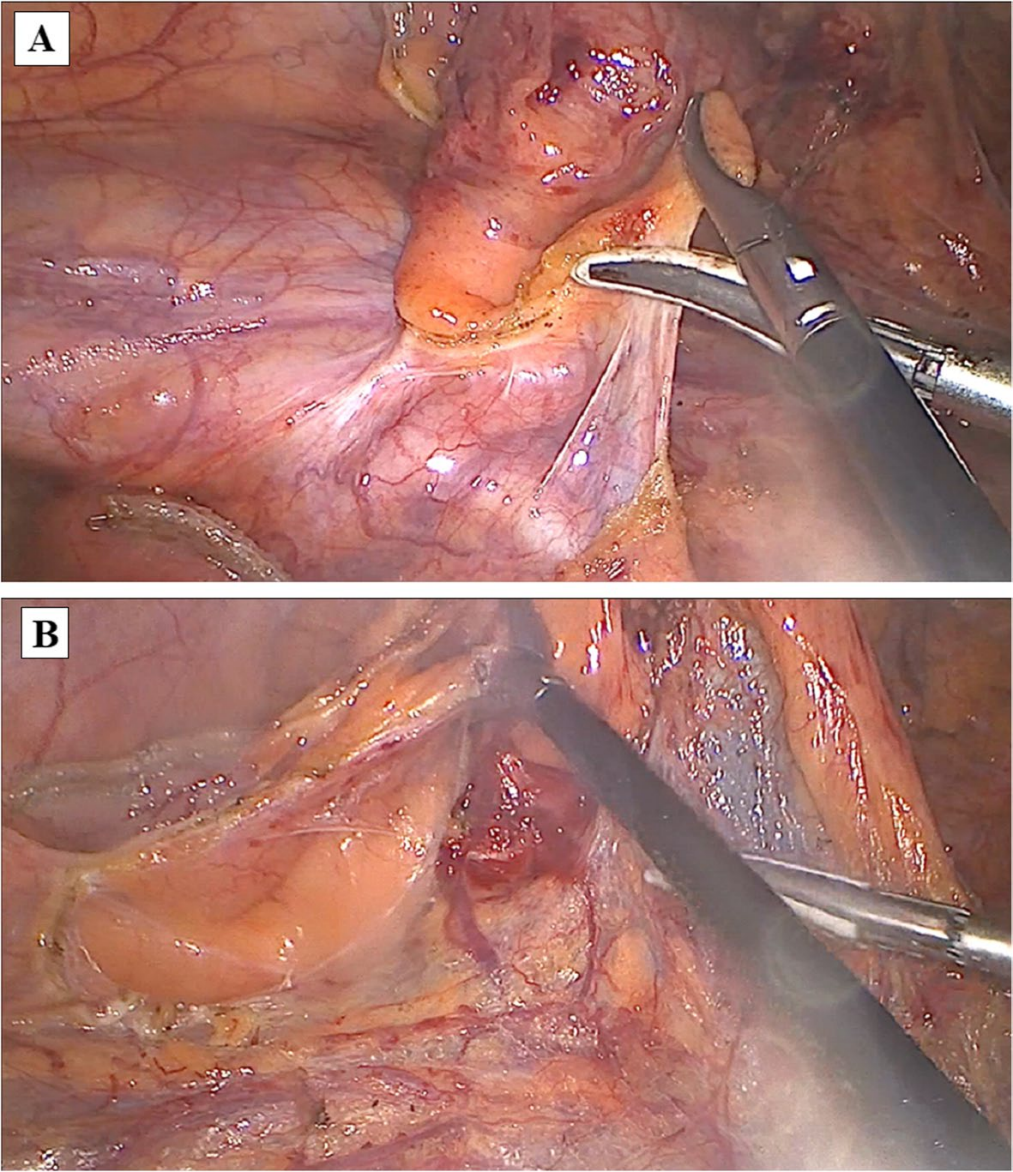
Open the mesenteric root peritoneum to enter the space between mesocolic fascia and the Toldt’s fascia in front of the Gerota fascia and then into the Fredet space

**Fig. 7:**
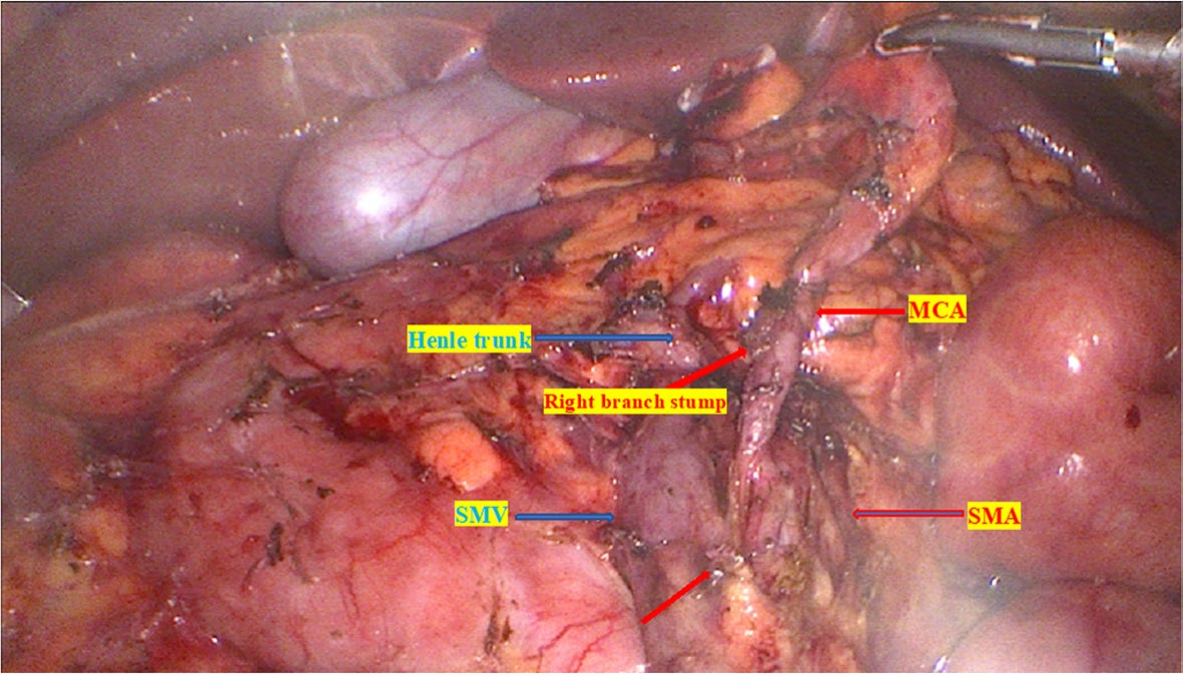
D3 lymphadenectomy and central vascular ligation (ICA, ileocecal artery; MCA, middle colic artery; SMA, superior mesenteric artery; SMV, superior mesenteric vein)

Step 3 (Caudal approach): Low head position, left side tilted, turn the terminal ileum and cecum upward, and open the mesenteric root peritoneum to enter the space between mesocolic fascia and the Toldt’s fascia in front of the Gerota fascia and then into the Fredet’s space (Fig. 6). From the opening mesenteric window at the terminal ileum (avascular area of Treves), open the retrograde mesenteric towards the origin of the ileocecal vascular, dissecte the lymph nodes around this artery, and ligate these artery and vein at their origin with LigaSure™ (central vascular ligation) (Fig. 7). Continue dissection up to both the anterior and posterior aspect of the superior mesenteric axis, the right colic artery root (if present), lymph node dissection (D3 lymphadenectomy), and radical ligation of the right colic artery (central vascular ligation) (Video 2). Dissect, dredged up around the origin of the middle colonic artery, dredged along this artery to the bifurcation, resect the right branch, then resect the transverse mesentery towards the transverse colon, then dissect it with a stapler, and cut the great omentum (gastrocolic ligament) 10 cm from the tumor. If the right-sided transverse colon to be removed was only within the blood supply boundary of the right branch of the MCA, we did not ligate the MCA but still removed the entire lymph node from the origin of the MCA up to the bifurcation of the right and left branches. The blood supply boundary was detected by dissection of the lymph nodes from the origin of the MCA upward. With cases of right-sided transverse colon tumors, extended right hemicolectomy was needed, and then in these cases, we dredged up and resected the MCA around its origin. Or in some cases, if the right-sided transverse colon resection encroaches on the blood supply boundary of the left branch of the MCA, or in cases of detecting suspicious positive lymph nodes around the left branch of the MCA, then the MCA was resected around its origin.

Step 4: Continue resecting caudally and medially to laterally the Toldt fascia of the colon to release the entire right colon from the abdominal wall (Video 2).

- *Stage 3*: Performing side-to-side or functional end-to-end anastomosis intra-abdominally by using a straight stapler combined with hand-stitching to close the hole. Close the mesentery with continuous stitches (Video 2).

**Fig. 8:**
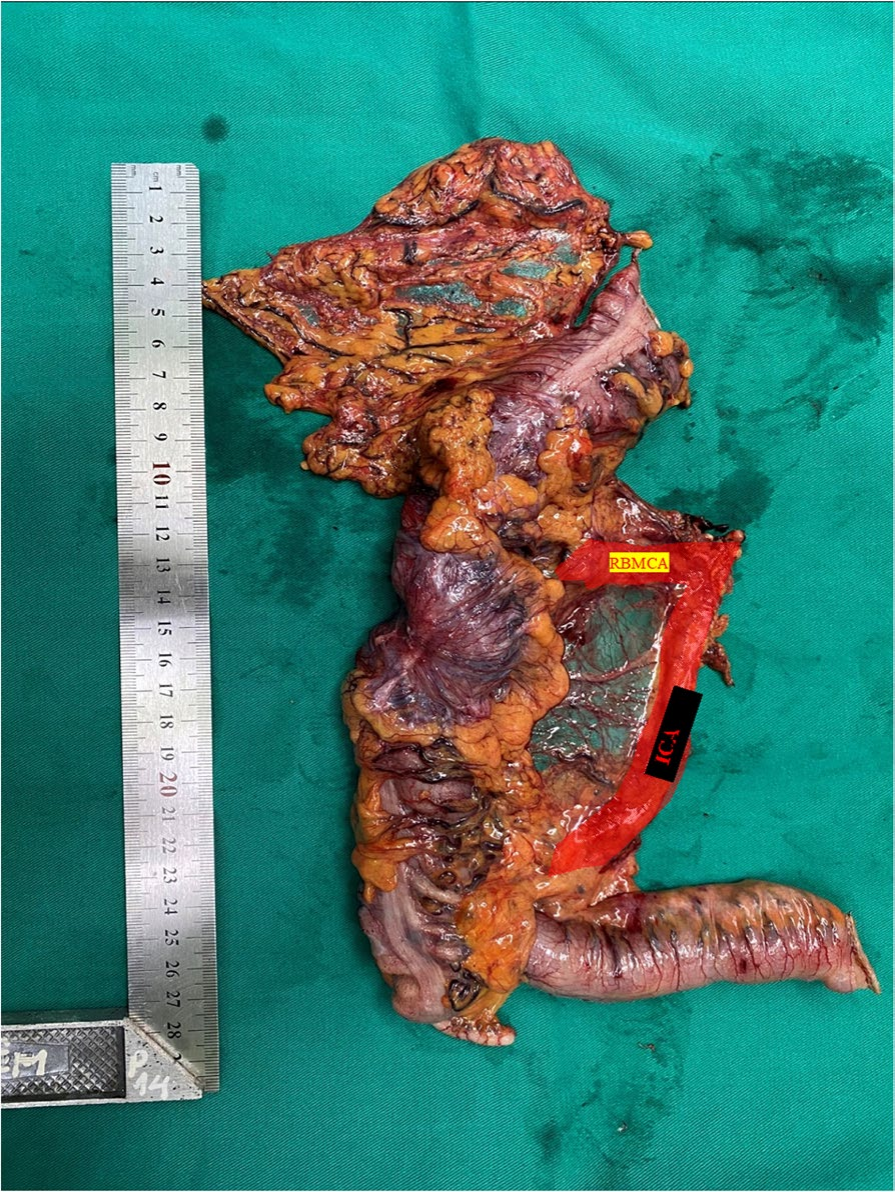
Specimen after tLRH_D3, CME/CVL_ as well as intact surgical trunk framing the peritoneal window (PW) between the stumps of the ileocecal artery (ICA) and the right branch of the middle colic artery (RBMCA)

If the tumor is in the colic hepatic flexure, it may be necessary to dissect the pyloric lymph nodes, ligation of the right gastroepiploic artery, and the extended right hemicolectomy. Collect the specimen through the small opening across horizontal line on pubic bone or through the natural orifice (vagina) (Fig. 8).

## Results

In January 2022 to December 2022 (12 months), there were a total of 32 cases of primary right-sided colon malignancies that have undergone tLRH_D3, CME/CVL_ based on the Bach Mai Procedure — a novel-combining approach and early resection of the terminal ileum. General and preoperative information as well as short-term outcomes (Table 1) were prospectively collected and analyzed.

**Table 1:** Demographic characteristics and postoperative short-time outcomes

Index	*N* (%)
**Sex ratio (M:F)**	16/16
**Age (years old, mean, min–max)**	61.03 ± 13.78 (30–84)
**BMI (kg/m**^**2**^**, mean, min–max)**	21.47 ± 2.35 (17.6–26.6)
**CEA (ng/mL, mean, min–max)**	5.12 (1.55–16.4)
**T stage**	
1	1 (3.1%)
2	4 (12.5%)
3	22 (68.8%)
4	5 (15.6%)
**AJCC**	
I	5 (15.6%)
II	17 (53.1%)
III	10 (31.3%)
IV	0
**Maximum diameter of tumor (cm) (median, min–max)**	5 (3–12)
**Tumor site**	
Cecum-ascending	29 (90.6%)
Hepatic flexure	3 (9.4%)
**Tumor’s differentiation**	
Well	9 (28.1)
Moderate	20 (62.5)
Poor	3 (9.4)
**Operation time (minutes) (mean, min–max)**	213.91 (160–330)
**Length of resected bowel (cm) (median, min–max)**	25 (15–47)
**Conversion**	0 (0%)
**Bleeding (ml) (median, min–max)**	70 (20–180)
**Postoperative stay (days)**	8 (7–9)
**LNN count (median, min–max)**	38 (22–101)
**Positive LNN count (median, min–max)**	1 (0–9)
**\Serious postoperative complications (grade 3 or higher)**	0
**Inhospital mortality**	0

Thirsty-two patients were included in the group, with 16 men and 16 women. The mean of age, BMI, and CEA serum level were 61.03 years old, 21.47 kg/m^2^, and 5.12 ng/mL, respectively. In 3 cases (9.4%), the tumor site was hepatic flexure, and the median tumor size was 5 cm. The mean of operative time and bleeding was 183.91 min and 70 ml. The median of lymph node number (LNN) was 38, with the maximum number which was 69. No serious postoperative complications (grade 3 or higher) neither inhospital mortality was detected. The median of postoperative stay was 6 days.

Pathologically, 100% of cases had R0 section. Twenty-two cases (68.8%) of tumors were at T3 stage, and there were correspondingly twenty-seven cases (84.4%) of tumors which were at stages 2–3 according to AJCC 8th Cancer Staging. There was one case of T1 stage, and in this case, the tumor location was preoperatively identified by autologous blood marking [19].

## Discussion

A multicenter study by Mario Guerrieri et al. found that the group of patients undergoing laparoscopic colectomy had a shorter hospital stay than the open surgery group, as well as lower medical costs, and lower complication and mortality rates [20]. Nelson H. et al. in a multicenter study are comparing two groups of colon adenocarcinoma patients randomized to laparoscopic or open surgery on 1134 with 3-year follow-up in 2 phases. The following results were available: overall survival after 3 years of follow-up was not different between the 2 groups; operative time in the laparoscopic group was longer than in the open surgery group (*p* < 0.001); postoperative recovery time as well as hospital stay in the laparoscopic group was shorter than in the open surgery group (*p* < 0.001); feeding time in the laparoscopic group was also earlier (*p* < 0.001); and the duration of analgesia in the laparoscopic group was also shorter (*p* < 0.001) [21]. Laparoscopic surgery in these cases still prevails due to the following:

- Access to blood vessels and lymph nodes to perform CME, CVL, and D3 lymphadenectomy is easier than open surgery, without touching the tumor and without having to pull the abdominal wall, so it is better in terms of oncology, and patients do not have pain due to manipulation of abdominal wall contraction postoperatively.
- The incision for specimen collection in laparoscopic surgery is always smaller than the open incision for large tumors.
- It is possible to choose the position of opening the abdominal wall to take specimen during laparoscopic surgery without affecting the surgery. It could be an old incision of the patient. If the patient has never had any abdominal surgery, the horizontal line on the pubic bone will be chosen due to the aesthetic, and the incision below the umbilical after surgery will have less effect on breathing as well as reducing pain, which is convenient for performing enhanced recovery after surgery (ERAS).

Unlike the proved benefits of total mesorectal excision (TME) in oncological and survival outcomes, complete mesocolic excision (CME) has recently developed and studied on right-sided hemicolectomy. The right colon was considered a peritoneal organ and lacked a mesentery, while Sir Treves has believed that the mesentery disappeared after integration of the left and right mesenteric fusion [22]. So, the CME concept was based on three main points: dissection in the embryological plane, central vascular ligation, and a sufficient length of resected bowel. Recently, in two systematic reviews, complete mesocolic excision has shown favorite results on long-term survival outcomes, but no differences in anastomotic leak rate or perioperative mortality [11, 23]. However, these reviews still have some limitations on the quality of evidence, which have no level 1 evidence from a randomized clinical trial (RCT) [11]. To date, there was just one prospective controlled trial to evaluating long-term outcomes of CME procedure, which was revealed that CME have improved the local recurrence-free survival (LRFS) significantly without improving postoperative complications [24]. A recent phase-3 randomized clinical trial RELARC for short-term outcomes of CME undergoing laparoscopic right hemicolectomy have shown the postoperative surgical complication rate was no difference, but the intraoperative complications, including vascular injury, were significantly higher in the CME group versus convenient group. So, CME in laparoscopic right hemicolectomy should be proceed by experienced surgeons [25].

In Japan, the randomized clinical trial JCOG0404 was performed comparing D3 lymphadenectomy laparoscopic versus open surgery in patients with stage 2 and stage 3 colon cancer from 2004 to 2009. In this trial, D3 lymphadenectomy is prescribed as a standard surgery for both laparoscopic and open surgery. The study had 524 patients undergoing open surgery and 533 patients undergoing laparoscopic surgery; the study results showed that patients with laparoscopic surgery had less intraoperative blood loss (*p* < 0.001), even though laparoscopic surgery lasted for more than 52 min (*p* < 0.001). The group of laparoscopic patients had a shorter transit time, less postoperative analgesia, and a shorter hospital stay. Complications were lower in the laparoscopic group (14.3%, *p* < 0.001). The study concluded that laparoscopic D3 lymphadenectomy initially gave safe results and had some clinical benefits [26, 27]. In our research, the mean of operative time was 213.91 min, which was quite similar with research by Jin-Tung Liang et al. [16] but longer than the research by DukYeon Hwang et al. [15]. No serious postoperative complications (grade 3 or higher) neither inhospital mortality was detected, and the median of postoperative stay was 6 days. Pathologically, the median of lymph node number (LNN) was 38, which was quite similar with research by Jin-Tung Liang et al. and DukYeon Hwang et al. [15, 16] and more superior with research by Songtao Du et al. and Gennaro Galizia et al. [28, 29]. This difference could be explained by in western; the concept of CME-CVL has not systematically include the resection of lymph nodes along the gastroepiploic artery including the infrapyloric area, while in the eastern, the JSCCR D3 dissection omits removal of the gastroepiploic and infrapyloric lymph nodes [13]. And the differences in the Bach Mai Procedure versus other procedures were coordinating different approaches (cranial, medial-to-lateral, and caudal approaches) and early resection of the terminal ileum. In our technique, a combining approach helped take advantage of each approach: a cranial approach helped identify and control important anatomical landmarks from the outset, such as the right colonic veins (in pure caudal-to-cranial approach, it was difficult to control the root, easy to tear from the body of Henle causing uncontrol bleeding endoscopically) and locate the origin of the middle colonic bundle as the upper landmark of the surgical axis, descending the transverse and hepatic flexure colon, thereby gaining access to the space between the mesenteric flexure of the liver and the fascia of Gerota and accessing Fredet’s space. Moreover, thanks to early ileostomy, access to the spaces between the right mesenteric colon and Toldt’s fascia, Gerota fascia, and Fretdet space becomes convenient (not “tunneling” like other techniques), rarely misclassifying dissection, and quickly met the dissection layer from the top down to release the entire right mesentery without touching the tumor; the dissection of lymph nodes around the surgical superior mesenteric axis and access to the right mesenteric root as well as the base of the vascular bundles were easy and quick and safer than other approaches, and there was no significant difficulty in having colonic vascular anatomical changes.

Vergis, Steigerwald, Bhojani, Sullivan, and Hardy compared laparoscopic right colectomy with intra-abdominal versus extra-abdominal anastomosis and found no difference in the rate of fistula or mortality after surgery. Otherwise, intra-abdominal anastomosis has several advantages versus extra-abdominal anastomosis: faster recovery of intestinal motility, shorter length of incision, more quick recovery of digestive function, lower rate of paralytic ileus, less postoperative analgesia, less complications grades 1 and 2 according to Clavien-Dindo classification, and lower rate of hernia through incision. However, intra-abdominal anastomosis prolongs surgery and increases costs [30]. In our study, the mean of time for performing anastomosis was 27.35 min, not so long compared to time of performing extra-abdominal anastomosis, so that this technique should be proceeded by experienced surgeons. In our study, there were no cases of conversion, and in all of cases, the anastomosis proceeded intra-abdominally without postoperative fistula.

## Conclusion

The Bach Mai Procedure — a novel-combining (cranial, medial to lateral, and caudal) approach with early resection of the terminal ileum, is technically feasible and safe for tLRH_D3, CME/CVL_. With limited resources and supporting tools, we have successfully implemented one of the most difficult surgical techniques of colon as well as gastrointestinal surgery. Further investigations and follow-up must be proceeded to evaluate the long-term outcomes of our technique.

## Supplementary Information

**Additional file 1:** **Video 1.** Cranial approach and Medial-to-lateral approach: Dissected the transverse colic mesentery and a part of the right mesentery from the anterior surface of the pancreatoduodenal junction to enter the Fredet's Fascia and exposed the surgical axis - the superior mesenteric vascular axis.

**Additional file 2:** **Video 2.** Caudal approach and Lateral-to-medial approach: Dissected the lymph nodes around ileocecal artery, and ligated this artery at its origin, continuing dissection up to both the anterior and posterior aspect of the superior mesenteric axis, lymph node dissection (D3 lymphadenectomy), resected the right branch of the middle colonic artery.

## Data Availability

All data generated or analyzed during this study are included in this published article.

## Abbreviations

AJCC: American Joint Committee on Cancer
CME: Complete mesocolic excision
CVL: Central vascular ligation
ICA: Ileocecal artery
LRH: Laparoscopic right hemicolectomy
MCA: Middle colic artery
SMA: Superior mesenteric artery
SMV: Superior mesenteric vein
RCC: Right colon cancer
RSC: Right-sided colon
